# Supplementary material for: Proportion of children aged 9–59 months reached by the 2017 measles supplementary immunization activity among the children with or without history of measles vaccination in Lilongwe district, Malawi
Source: PLoS One. 2021 Jan 11;16(1):e0243137. doi: 10.1371/journal.pone.0243137 (PMC7799760; doi:10.1371/journal.pone.0243137)
Supplement: S1 File — An original interviewer-administered questionnaire which was later transferred to Open Data Kit (ODK) to be ectronically administered. These questions were developed specifically for this study. (DOCX) [file pone.0243137.s001.docx]

**1.3. QUESTIONNAIRE**

**Missed opportunities for measles vaccination among children during the June 2017 measles Supplementary Immunization Activity in Lilongwe district, Malawi.**

Date of interview……………………. Study ID…………………………………

Name of investigator……………………………………………………………………...

Starting time…………………………. Ending time……………………….

1. **Child’s demographics**

| **Item** | **Question** | **Responses** |
| --- | --- | --- |
| R_10 | Sex of child | 1: Male  2: Female |
| R_11 | Child’s age (in months)  Ask the date of birth |  |
| R_12 | Child’s birth order | 1: 1^st^  2: 2^nd^ – 4^th^  3: ≥5th |
| R_13 | Child’s place of delivery | 1: Home  2: H/Facility |

1. **Primary caretaker’s socio-demographics**

| **Item** | **Question** | **Responses** |
| --- | --- | --- |
| R_14 | Caretaker’s age (in years).  Ask the date of birth |  |
| R_15 | Relationship to child | 1.Biological parents  2.Non-biological parents  3.Any relative |
| R_16 | Marital status | 1 .Single  2. Married  3. Widowed  4. Divorced /separated |
| R_17 | Education level | 1. No education  2. Primary  3. Secondary  4. Post-secondary |
| R_18 | Employment status | 1. Unemployed  2. Self employed  3. Casual laborer  4. Professional  5. Housewife |
| R_19 i | Frequency of reading newspaper | 1.Not at all  2. less than once a week  3.At least once a week |
| R_19ii | Frequency of listening to radio | 1.Not at all  2. less than once a week  3.At least once a week |
| R_19iii | Frequency of watching television | 1.Not at all  2. less than once a week  3.At least once a week |

1. **Household factors**

| **Item** |  |  |
| --- | --- | --- |
| **R_20** | Sex of household head | 1. Male 2. Female |
| R_21 | Head of household | 1. Child headed 2. Woman headed 3. Man headed |
| R_22 | Age of head of household  Ask date of birth |  |
| R_23 | Education level of household head | 1. No education  2. Primary  3. Secondary  4. Post-secondary |
| R_24 | Employment status of household head | 1. Unemployed  2. Self employed  3. Casual laborer  4. Professional |
| R_25 | Number of under 5 year old children in household |  |
| R_26 | Religious affiliations | 1.Orthodox  2. Muslim  3. Protestant  4. other specify____________________ |

1. **Routine immunization data**

| **Item** | **Question** | **Response** |
| --- | --- | --- |
| R_27 | Has the child ever received an injection on the left upper arm? That is measles injection at the age of 9 months or older at health facility - to prevent him/her from getting measles. | 1: Yes  2: No  99: Do Not Know |
| R_28 | How many times was measles vaccine given at a health facility? | Number  99: Do Not Know |
| R_29 | Do you have a vaccination card | 1. Yes  2. No |
| R_30 | If vaccination card not available, ask caretaker to state child’s age (in months) when the child received MCV1, and where? | 99: Do not know |
| R_31 | If not, why was the child not vaccinated against measles? | 1.vaccine safety  2. child was sick  3.measles not a big  threat as other diseases  4.Religious beliefs  5.Distance to H/Facility  or vaccination post  6.Too long waiting time at the facility |

1. **Measles supplementary immunization activity**

| **Item** | **Question** | **Response** |
| --- | --- | --- |
| R_32 | Did the child participate in 2017 measles rubella campaign? | 1. Yes  2. No |
| R_33 | Do you have any evidence or vaccination card that shows the child was vaccinated during the SIA?  *If caretaker is unable to produce card, probing questions will be asked to verify the vaccination history of the child* | 1: Yes  2: No |

**The following questions only apply if the child did not participate in the 2017 measles campaign**

| R_34 | If not, what was the reason for child not to participate? | 1.vaccine safety  2. child was sick  3.measles not a big threat as other diseases  4.Religious beliefs  5.Distance to vaccination post  6. Too long waiting time |
| --- | --- | --- |
| R_35 | Did the health authorities provide with sufficient information about the recent measles-rubella immunization campaign? | 1: Yes  2: No  99: Do not know |
| R_36 | Do you feel you got enough information about measles vaccine and its safety? | 1: Yes  2: No  99: Do not know |
| R_37 | Do you remember any event/s in the past that would discourage from getting your child vaccinated against measles? | 1: Yes  2: No |
| R_38 | Can you name the event in the past that diminished your trust in measles vaccination |  |
| R_39 | Do you consider that some vaccines are more important than measles? | 1: Yes  2: No  99: Do not know |
| R_40 | If yes, why do you think so? |  |
