# Supplementary material for: Proportion of children aged 9–59 months reached by the 2017 measles supplementary immunization activity among the children with or without history of measles vaccination in Lilongwe district, Malawi
Source: PLoS One. 2021 Jan 11;16(1):e0243137. doi: 10.1371/journal.pone.0243137 (PMC7799760; doi:10.1371/journal.pone.0243137)
Supplement: S2 File — This guide was used to collect qualitative data through Key Informant Interviews. (DOCX) [file pone.0243137.s002.docx]

**KEY INFORMANT INTERVIEW GUIDE**

| Respondent code | Date  (dd/mm/yy) | Interview number |
| --- | --- | --- |
|  |  |  |

**To be completed by the interviewer**

**READ THE INFORMED CONSENT FORM TO THE RESPONDENT AND ASK THEM TO SIGN IT.**

| 1. **Introduction** |
| --- |

This interview aims to identify health system factors that were associated with vaccination and /or non-vaccination of eligible children during the June 2017 measles SIA in Lilongwe district.

It will focus on thematic areas that are critical to health system building blocks framework; thus financing, human resource, logistics, service delivery and information system.

- 1. What is your current position? (WRITE THE RESPONSE BELOW)

|  |
| --- |

- 1. In what capacity did you participate in June 2017 measles-rubella SIA in Lilongwe district? (WRITE THE RESPONSE BELOW)

|  |
| --- |

- 1. Could you please briefly explain how the measles mass campaign was planned and implemented
  2. How was the information passed to the masses to bring their children to various vaccination posts for measles immunization

(Probe further in areas of HIS)

- 1. What were the challenges that were experienced during the campaign?

(Probe further in areas of interest.)

- 1. Some children missed the opportunity to be vaccinated against measles during the June 2017

Measles SIA. In your opinion, what do you think were the reasons for non-vaccination from the health system perspective?

(Probe on financing, human resource, logistics.)

- 1. In your opinion, how do you think the SIA should have been planned and implemented so that eligible children are not missed?
