# Supplementary material for: Proportion of children aged 9–59 months reached by the 2017 measles supplementary immunization activity among the children with or without history of measles vaccination in Lilongwe district, Malawi
Source: PLoS One. 2021 Jan 11;16(1):e0243137. doi: 10.1371/journal.pone.0243137 (PMC7799760; doi:10.1371/journal.pone.0243137)
Supplement: Attachment — Submitted filename: PLOS_TA.docx [file pone.0243137.s003.docx]

Summary

There is great value in exploring reasons behind missed opportunities for vaccination (MOV). Since the 1980s, the WHO has had several iterations of [methodology for assessing MOV](https://apps.who.int/iris/bitstream/handle/10665/259201/9789241512954-eng.pdf?sequence=1). Since MOV is defined in the context of health systems failing to vaccinate an individual eligible for a vaccine, these tools are designed in the context of facilities. Moreover, MOV and supplementary immunization activities (SIA) are separate concepts where SIAs complement routine vaccination. This makes it rather confusing that the proposed study for publication explores MOV in the context of SIA. Individuals not reached through SIAs would not be MOV. If the focus of the study is to assess why children may not have been reached by the measles SIA, this would be different than examining MOV. There are issues with both the design and analysis proposed in the paper.

Specific edits

*Background*

- The WHO defines missed opportunities for vaccination (MOV) as an individual making contact with the health system and not receiving a vaccination they are eligible for. [Supplementary immunization activities (SIA) are intended to complement routine vaccination and are not specifically considered a WHO strategy specifically for addressing MOV](https://apps.who.int/iris/bitstream/handle/10665/330101/9789241516310-eng.pdf?ua=1). These need to be clearly defined if the paper is framed in the context of MOV and SIA.
- Revisit literature on gaps in reaching children during mass measles vaccination coverage. There is documentation on how SIAs miss marginalized populations/gaps in coverage.
- Revisit citations 4-10 – these primarily look at reasons for MOV in the context of regular vaccination not people being missed through SIA.
- The background should include more robust information on measles coverage (and source) in Malawi and Lilongwe. The paper cited, which were vaccine coverage estimates based on surveillance data, is not the most appropriate data source compared to household survey and DHIS2 in Malawi. In Malawi, a DHS was conducted in 2015/16 and a SPA was conducted in2013/14. The [Malawi progress report to Gavi](https://www.gavi.org/sites/default/files/document/joint-appraisal-malawi-2018pdf.pdf), which includes the measles campaign cites DHS and DHIS2 data to describe measles coverage and measles surveillance data. DHIS2 data in Lilongwe during the SIA campaign period should be cited.
- It’s especially surprising that SPA findings are omitted from the paper given SPA has indicators directly related to MOV.

*Methods*

- Why was measles SIA not captured on vaccination cards? Recall by vaccination card is considered the best practice for determining vaccination coverage in a household survey and is preferable over self-reported recall. The [WHO MOV guidelines specifically advise to not accept verbal vaccination recall from mothers](https://apps.who.int/iris/bitstream/handle/10665/259201/9789241512954-eng.pdf?sequence=1).
- Insufficient justification why only eight KIIs were conducted. Were KIIs all with public health facility providers or CHAM/NGOs as well?
- Insufficient justification on how the IDI guides for both parents and health workers deviate from MOV suggested protocol and standard question approaches with core omitted questions such as “Has this child ever been vaccinated?” Also would be important to describe the choice of choosing a prompted approach for asking “why was the child not vaccinated against measles” instead of an unprompted approach since this is a core question for the study.
- Asking about sex of household head is different than asking who makes decisions in a household (preferred approach).
- Based on question wording in the KII guide, there is a potential that respondents may have confused campaign-related factors with health system-related factors that would impede in vaccination.

*Results*

- It is unclear how the authors define “missed the opportunity of receiving measles vaccine.” The results should cite the total number of children considered “eligible” or in need of vaccination and clearly define the denominator used for coverage estimates.
- As described above, “MOV” should not be used to describe children that were not reached by the SIA campaign.
- The qualitative interviews could be more robustly and systematically analyzed. For example, issues with the health information system are cited in the discussion as a major finding. However, the quote used to support the health information system is about flawed use of population estimates – which is independent of the health information system. Was the health officer referring to DHIS2 or census estimates? I believe DHIS2 sits with the Ministry of Health in Malawi, not the National Statistical Office, so the respondent could have been referring to just census estimates (not the health information system).

*Discussion*

- As described above, “MOV” should not be used to describe children that were not reached by the SIA campaign.
